# CD19-negative relapse after CAR-T cell therapy: mechanisms of antigen escape and lineage switch

**DOI:** 10.3389/fimmu.2026.1692287

**Published:** 2026-04-07

**Authors:** Jiawen Huang, Xiaobing Huang, Duonan Yu

**Affiliations:** Department of Hematology, Sichuan Academy of Medical Sciences and Sichuan Provincial People's Hospital, University of Electronic Science and Technology of China, Chengdu, China

**Keywords:** CAR-T cell therapy, CD19^-^ relapse, leukemia, loss of antigen, lymphoma

## Abstract

CD19 chimeric antigen receptor (CAR)-T cell therapy has transformed the treatment of relapsed/refractory B-cell malignancies, achieving high remission rates. Nonetheless, 20%-40% of patients eventually relapse, classified as either CD19^+^ or CD19^−^ relapse. Most relapses are CD19^+^, largely due to reduced potency and poor persistence of CAR-T cells after infusion. In contrast, a substantial fraction of patients develops CD19^−^ relapse driven by selective pressure from CAR-T cell therapy. Post-CAR-T cell CD19^−^ relapse primarily arises through three mechanisms. First, pre-existing CD19^−^ subclones, particularly those with progenitor-like features, expand because of their survival advantages. Second, CD19 expression is downregulated through pre-existing or newly acquired genetic alterations (mutations and abnormal splicing), epigenetic silencing, and post-transcriptional dysregulation. Third, lineage switching to a myeloid phenotype increases notably after CAR-T cell treatment. This myeloid conversion is frequently associated with KMT2A rearrangements and is driven by epigenetic reprogramming, impaired expression of B-cell regulators, and the presence of bipotent progenitors within leukemia or lymphoma. CD19^-^ relapse constitutes a major clinical barrier to the durable efficacy of CD19-CAR T-cell therapy. Understanding these diverse escape mechanisms is crucial for developing preventive strategies and effective salvage therapies to maintain long-term remission. To mitigate CD19^-^ relapse, a coordinated dual strategy is needed. First, novel therapeutic approaches should be developed, including CAR products targeting alternative antigens, optimized salvage regimens, and CD19 re-induction strategies. Second, high-resolution molecular profiling should be used to identify high-risk patients and guide pre-emptive or combinatorial interventions based on the molecular underpinnings of CD19^-^ relapse. Together, these approaches will deepen mechanistic insight into CD19^-^ relapse and enable targeted prevention and management of this key limitation of CAR T-cell therapy.

## 1 Introduction

CD19-directed chimeric antigen receptor (CAR) T-cell therapy has revolutionized the treatment of B-cell malignancies, achieving high initial response rates in patients with B-cell acute lymphoblastic leukemia (B-ALL) and diffuse large B-cell lymphoma (DLBCL) (**Table 1**). Real-world data from the Center for International Blood and Marrow Transplant Research (CIBMTR) on 410 pediatric and young adult (CAYA) patients treated with commercial tisagenlecleucel showed an initial complete response (CR) rate of 85.5% for ALL, and 39.5% for non-Hodgkin lymphoma (NHL) (54). However, durable remission remains a challenge, with 20%-40% of patients eventually relapsing (44, 55).

**Table 1:** Summary of clinical trials and real-world studies which examined CD19 expression upon relapse/progression.

Study	Disease	Number of patients	Response	OS	EFS	RFS/ PFS	Relapse/progression (based on responders)				CD19 detected by
							Total	CD19^+^	CD19^−^	Unknown	
ELIANA (1) (updated in (2) with no CD19 expression)	CAYA with r/r B-ALL	75	81% (45 with CR; 16 with CRi)	90% (6mo); 76% (12mo)	73% (6mo); 50% (12mo)	80% (6mo); 59% (12mo)	22	1	15 (3 partial CD19^+^)	6	Flow cytometry
ELINA (3-year follow-up) (3)		79	82%	63% (3yr)	Median: 24mo; 44% (3yr)	48% (3yr)	33	2	19 (3 partial CD19^+^)	12	
							9 with late relapse (>1yr)	2	5	2	
ZUMA-1 (4–7) (only (4) of phase 2 reported CD19 expression; phase 1 only enrolled 7 patients (8))	Adults with refractory LBCL	101 (77 DLBCL; 24 PMBCL/TFL)	83% (58% CR)	42.6% (5yr); Median: 25.8mo	43% (1yr); 38% (2yr); Median: 5.7mo (in (6))	DFS: 51% (5yr)	11 evaluated (with CD19^+^ at baseline) (59 in follow-up (7), no details reported)	8	3	0	IHC
ZUMA-2 (9, 10)	Adults with r/r MCL	68	91% (68% CR)	Median: 46.6mo; 83% (in 1yr follow-up)		Median:25.8mo; 61% (in 1yr follow-up)	14 (10) (32 in 3yr follow-up, no details reported)	13 (10)	1 (10)	0	IHC
ZUMA-3 (phase 1) (11)	Adults with r/r B-ALL	45 (with 3 different doses)	69% (53% CR, 16% CRi);	Median: 12.1mo		Median: 7.3mo	13	7	3	3	Flow cytometry
ZUMA-3 (phase 2) (12, 13)	Adults with r/r B-ALL	55	71% (56% CR, 15% CRi);	25.4mo;		Median:11.7mo; 42% (18mo)	13 (only reported in (12))	6 (12)	3 (12)	4 (12)	Flow cytometry; IHC for dry tap
ZUMA-5 (14–16)	Adults with indolent NHL	159 (127 FL, 1 DLBCL, 31 MZL)	90% (75% CR)	69.0% (60mo)		Median: 62.2mo	14 evaluable (as in (16), 13FL, 1MZL)	14 (all CD19^+^CD20^+^);	0 (but 2 CD19^−^ relapse from NR (14))	>24 (only reported 38 for FL (16))	IHC (CD19^+^ relapse was confirmed by flow cytometry)
(17, 18) ( (19, 20) is part of this study)	CAYA with B-ALL and DLBCL	52 (50 B-ALL; 2 DLBCL)	62% CR for B-ALL (28 with MRD^−^ CR, no results for DLBCL)	Median: 10.5mo	Median: 3.1mo; 52.0% (3mo); 38% (6mo)		8 (17)	2	5 (including CD19-dim)	1	Flow cytometry
							Latest follow-up: 9 (2 after HSCT + 7 with no HSC) (18)	3	3 (contradict with (17))	1 + 2 (Not reported for the 2 after HSCT)	
(21, 22)	CAYA with r/r CD19^+^ALL	43	MRD^−^ CR: 93%	69.5% (12mo)	50.8% (12mo)		18	11	7 (1 LS)	0	Flow cytometry
(23)	Adults with r/r CD19^+^ B-ALL	30 (29 evaluable)	93% BM remission (86% CR)				9	7	2 (1 LS)	0	Flow cytometry
(24) ( (25) is part of this study)	Adults with r/r DLBCL or FL	38 (14 FL, 24 DLBCL)	RR: 65.8%; CR:55.3%			31% for DLBCL; 43% for FL (5yr)	12	11	1	0	Before: IHC; After: IHC and flow cytometry
(26)	Adults with r/r CLL	14	RR: 57% (4 CR, 4 PR)	Median: 29mo; 71% (18mo)		Median: 7mo; 28.6% (18mo)	4 (all PR cases),	1 (CLL at abdominal lymph nodes)	1 (as CD19-dim DLBCL)	2	Flow cytometry (even for lymph node biopsy)
(27, 28) ( (29–31) are related)	CAYA with CD19^+^ ALL (including CD19^+^ T-ALL)	59 ( (29) has 25 CAYA and 5 older adults)	CR: 93% (including one with CD19^+^ T-ALL)	79% (12mo)		76% (6mo); 55% (12mo)	20	7	13	0	Flow cytometry
Pedi CART19 (29)* ( (27, 28) are its follow-up, it is listed here as it provided unique details)	Patients with r/r ALL	30 (25 CAYA and 5 older adults)	CR: 90% (including 3 patients with low MRD)	78% (6mo)	67% (6mo)		7 (one AML after CAR-T with the shared cytogenetics as original B- ALL, but not reported as relapse)	4	3	0	Flow cytometry
13BT022 (32) ( (33) is part of this study)	CAYA with r/r B-ALL or B-LLy	74 (72 B-ALL; 2 B-LLy); as two cohort: CAR-retreat (n=33) and CAR-naive (n=41)	Naive: 98% RR (100% in B-ALL) e;	Naïve: 90% (12mo); 88% (24mo)	Naïve: 82% (12mo); 72% (24mo);	Naïve: 84% (12mo); 74% (24mo);	Naïve:12	Naïve:6	Naïve: 6 (2 partial CD19^+^);	0	Flow cytometry
			Retreat: 64% RR	Retreat: 76% (12mo); 55% (24mo)	Retreat: 47% (12mo); 37% (24mo)	Retreat: 74% (12mo); 58% (24mo)	Retreat: 8	Retreat: 7	Retreat:1 (CD19^+^ with minor CD19^−^)	0	
ENSIGN (34)	Children with r/r ALL	29	RR: 69%;	75.7% (6mo)		66.4% (6mo)	8	6	2	0	
(35)	Patients with r/r B-ALL	23	RR: 82.6% (52.2% CR)				7	5	2	0	Flow cytometry
16CT022 (36)	CAYA with r/r CD19^+^ B-ALL	70 (as high (≥ 40%) or low (< 40%) tumor burden cohort)	RR: 97% (77% CR, 14% CRi)	High: 67% (12mo), 60% (24mo);	High: 42% (12mo), 34% (24mo);		19	7	12 (1 partial CD19^+^, 2 LS)	0	Flow cytometry
				low: 96% (12mo), 92% (24mo)	low: 86% (12mo), 78% (24mo)						
(37)	CAYA with r/r CD19^+^ B-ALL	185 (184 evaluable)	85% CR	85% (6mo); 72% (12mo)	62% (6mo); 50% (12mo)		57	30	22 (3 LS)	5	Flow cytometry
(38)	CAYA with r/r B-ALL	25 (24 evaluable)	RR: 75%				4	4	0	0	Before: morphology and/or flow cytometry After: flow cytometry
(39)	CAYA with r/r B-ALL	420 (412 evaluable; 77 received prior blina)	CR: 91.3%	Median: 49.1mo; 65.1% (24mo)	Median: 20.8mo; 47.4% (24mo)	Median: 40.2mo; 56.4% (24mo)	161	69	90 (9 LS, 3 CD19-dim and 10 partial CD19^+^)	2	Flow cytometry
(40)	Patients with r/r B-ALL	166 (34 have been reported (1, 29))	11 failed to achieve MRD^−^ remission (with 7 NR)				67 (including MRD and overt relapse)	26	41 (2 partial CD19^+^, 3 LS)	0	Flow cytometry
(41)	Adults with r/r B-ALL	57 (53 evaluable)	RR: 85% (which achieved MRD^−^ CR)	Median OS for responder: 20mo	Median EFS for responder: 7.6mo		22	9	11 (5 with CD19-dim, 1 LS)	2	Flow cytometry
(42)	Adults with r/r B-ALL	20	RR: 85% (which achieved MRD^−^ CR)	69.1% (6mo); 63.8% (12mo); 58% (24mo)	68.3% (6mo); 48.3% (12mo); 48.3% (24mo)		8 (including MRD and overt relapse)	3	5 (1 at MRD level; the other 4 had no myeloid features)	0	Flow cytometry
(43)	Children with R/R ALL	24	RR: 83.3% (18 were MRD^−^)	63% (1yr); 42% (3yr)	6% (1yr); 37% (3yr)		10	9	1	0	Flow cytometry
(44)	Adults with r/r B-ALL	53	CR: 83% (32 with MRD^−^ CR)	Median: 12.9mo	Median: 6.1mo		25	21	4 (all from MRD^−^ CR patients)	0	Flow cytometry
(45)	CAYA with r/r B-ALL	35	RR: 86% (20 with MRD^−^)	56% (3yr)	Median: 17mo; 41% (3yr)		12	11	1	0	Flow cytometry
(46)	Patients with r/r B-ALL	20	RR: 90%	Median: 12.91mo		Median: 6.93mo	8	5	3	0	Flow cytometry
(47)	CAYA with r/r BCP-ALL	51	CR: 96% (41 with MRD^−^)	74% (18mo)	44% (18mo)		22	12	8	2	Flow cytometry
(48) (CD19 CAR-T cells with IL-6 knockdown)	Adults with r/r B-ALL	17	RR: 78.6%			Median: 22.2mo	4	2	1	1	Flow cytometry
(49)	Adults with r/r B-ALL	48	RR: 85.4% (35 CR, 6 CRi)	72.1% (12mo): 55.2% (24mo)		Median: 12.4mo; 54.5% (12mo): 35.8% (24mo)	11 evaluable	4	7 (2 partial CD19^+^)	0	Flow cytometry
(50)	Children with r/r B-ALL	50	RR: 98% (48 with MRD^−^)	74.9% (5yr)		67.8% (5yr)	15	11	4	0	Flow cytometry
(51)	CAYA with r/r B-ALL	115 (67 r/r and 48 MRD re-emergent CD19^+^)	CR: 100% (96.5% with MRD^−^)	70.7% (4yr)		68.7% (4yr)	37	16	10 (1 LS)	11	Flow cytometry
(52)	Adults with r/r B-ALL	23	RR: 84% (64% with CR/CRi)	Median: 23mo; 29% (4yr)		Median: 5.5mo; 17% (4yr)	10	8	1 (partial CD19^+^)	1	Flow cytometry
(53)	Patients with r/r B-ALL	51 (42 r/r and 9 MRD^+^ CD19^+^)	CR/CRi: 90% for r/r, 100% for MRD^+^				11 (2 with HSCT)	3	6 (including CD19^dim^)	2 (not reported for the 2 with HSCT)	Flow cytometry

Key studies or studies with large cohort (>40) are included. If a study includes multiple reports, information is derived from the latest one (unless specifically indicated which are cited after the number). OS, overall survival; EFS, event-free survival; RFS, relapse-free survival; PFS, progression-free survival; CR, complete response; Cri, CR with incomplete hematologic recovery; LS, lineage switch; LBCL, large B-cell lymphoma; PMBCL, primary mediastinal B-cell lymphoma; TFL, transformed follicular lymphoma; NR, no-response; MZL, marginal zone lymphoma; Children and young adults: CAYA; MCL, Mantle-Cell Lymphoma; DFS, Disease-specific survival (excluding deaths unrelated to disease progression); NHL, Non-Hodgkin Lymphoma; FL, follicular lymphoma; DLBCL, diffuse large B-cell lymphoma; MRD, minimal residual disease; HSCT, hematopoietic stem cell transplantation; IHC, immunohistochemistry; CLL, chronic lymphocytic leukemia; B-LLy, B-lymphoblastic lymphoma; blina, blinatumomab; BCP-ALL, B cell progenitor ALL.

Relapses are broadly categorized as CD19^+^ or CD19^−^. Reported evaluable cases (excluding unknowns) indicate that 54.5% of relapses are CD19^+^ and 45.5% are CD19^−^ (including CD19^dim^ and partial CD19^−^; **Table 1**). CD19^−^ relapse is notably more frequent in leukemia (47.5% of evaluable cases) than in lymphoma (9.8% of evaluable cases) (**Table 1**). CD19^+^ relapse is primarily associated with CAR-T cell dysfunction (56), and many strategies have been explored to address this issue (57), CD19^−^ relapse represents a distinct form of antigen escape, in which tumor cells evade immune recognition by downregulating or losing CD19 expression (1, 21, 44).

To comprehensively address this critical barrier, this review is divided into two parts. Part I​ examines the biological mechanisms​ underlying CD19 loss, ranging from pre-existing CD19^-^ clones to transcriptional regulation and lineage switching. Part II​ translates these insights into clinical practice, discussing current diagnostic approaches, therapeutic interventions, and future directions for preventing and managing CD19^-^ relapse.

## 2 Part I: mechanisms of CD19^−^ relapse after CAR-T cell therapy

### 2.1 Relapse due to pre-existing CD19^−^ B-cell clones

Pre-existing CD19^−^ malignant clones provide a direct explanation for CD19^−^ relapse and treatment failure. Tumor cells that lack CD19 can evade targeted therapies and cause antigen-negative relapse. Although malignant B-cells usually express CD19 (58, 59), minor CD19^−^ clones are detectable. In a retrospective analysis of 501 B-ALL cases, about 17% of patients had more than 1% CD19^−^ leukemic blasts before immunotherapy (60). CD19^−^ compartments may be enriched for leukemia stem cells (LSCs). Castor et al. (61) identified CD34^+^CD38^−^CD19^−^ cells with stem/progenitor features in t(12;21) and BCR-ABL1 ALL. CD34^+^CD38^−^CD19^−^ cells from Philadelphia chromosome-positive (Ph^+^) ALL patients differentiated and reacquired lineage markers in NOD/SCID mice, supporting their LSC potential (62). Likewise, only the CD34^+^CD10^−^CD19^−^ fraction from about 30 B-ALL patients maintained long-term *in vivo* proliferation (63). Stem cell-like CD34^+^CD19^−^ (64, 65) and CD133^+^CD19^−^ (66) subsets have been described in pediatric B-ALL, including rare CD19^−^ B-ALL (67). Additional CD19^−^ or CD19^dim^ B-ALL cases, some with severe bone damage and aleukemic presentation, have also been reported (68–70).

CD19 loss also occurs in other B-cell malignancies. CD19^−^ DLBCL has been documented (71, 72). In one case, uniparental disomy produced a homozygous mutation at the intron 3 acceptor site of *CD19* (71), causing intron retention, a premature stop codon, and loss of CD19 expression. Aberrant *CD19* splicing is a frequent feature of CD19^−^ relapse after CD19 CAR-T cell therapy and partly explains CD19 loss (discussed later).

**Figure 1:**
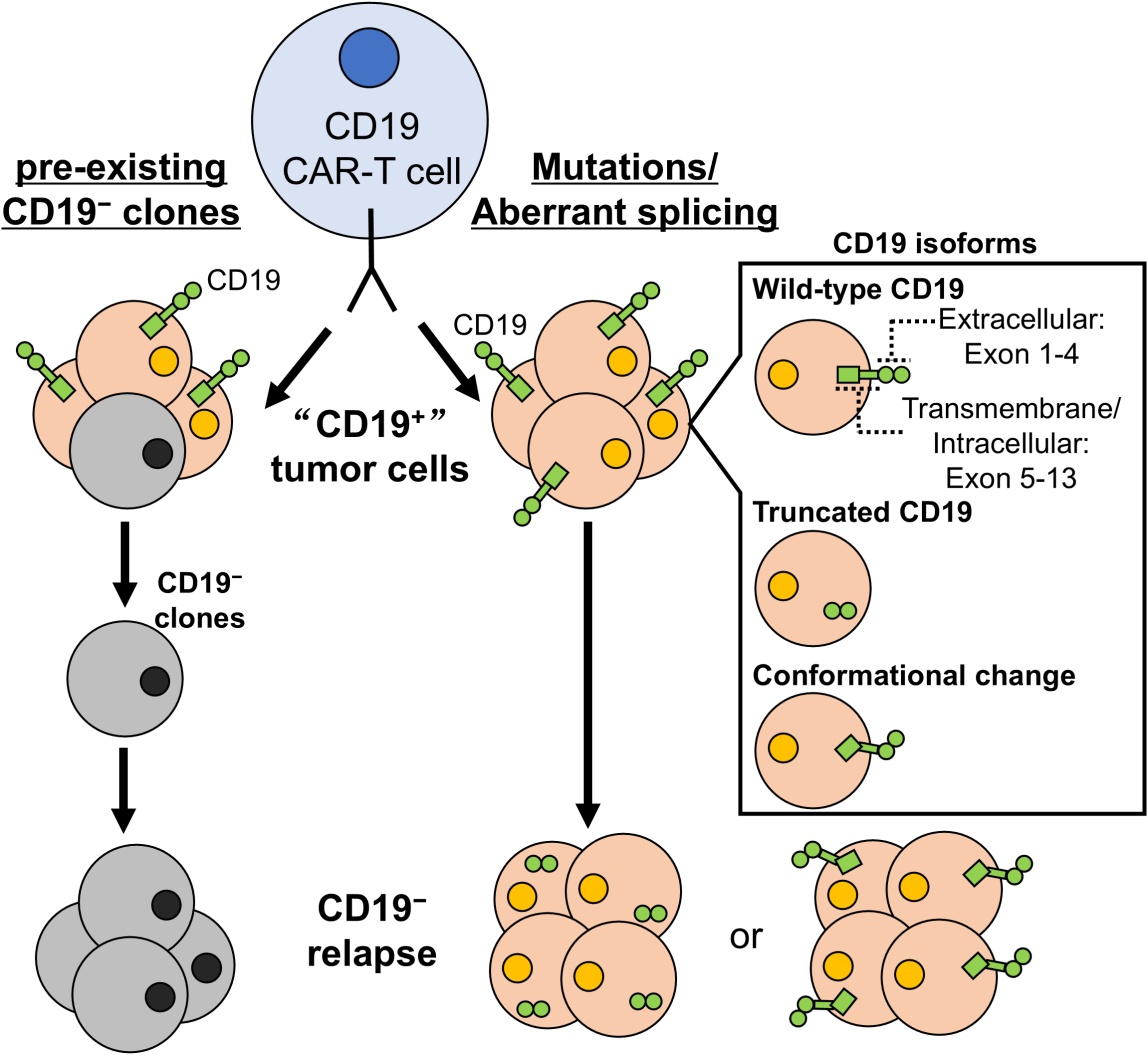
Expansion of novel subclones under selective pressure from CAR-T cells. CD19^−^ subclones pre-exist before CD19 CAR-T cell treatment. Under selective pressure from CAR-T cells, these subclones gain a survival advantage and expand, driving CD19^−^ relapse. Similarly, subclones expressing variant CD19 isoforms exist prior to treatment. These isoforms arise from mutations or aberrant splicing, generating truncated protein or proteins with altered conformation. Under selective pressure, subclones expressing specific CD19 isoforms, whether pre-existing or newly acquired, ​​escape CAR-T cell recognition. Expansion of these subclones also contributes to7 CD19^−^ relapse.

These CD19^−^ subpopulations, together with their progenitor properties, likely contribute to CD19^−^ relapse after CD19-directed treatment. Using single-cell profiling, Rabilloud et al. (73) showed that pre-existing CD19^−^ clones, only 0.03% of live cells, can drive CD19^−^ relapse after CAR-T cell treatment. Grupp et al. (74) linked baseline CD45^dim^CD34^+^CD19^dim^ subclones in ALL to rapid CD19^−^ relapse as early as day 23 post-infusion. Bueno et al. (75) similarly found that pre-existing CD34^+^CD22^+^CD19^−^ leukemic progenitors expanded markedly after CD19 CAR-T cell therapy, promoting phenotypic escape. In a trial of the CD19 CAR-T cell product axicabtagene ciloleucel for r/r large B-cell lymphoma (NCT02348216), about 30% of patients developed CD19^−^ relapse (76), driven by cells with very low CD19 expressions rather than CD19 mutations (**Figure 1**).

Although CD19^−^ subclones are central to evaluating CD19 CAR-T cell therapy efficacy and MRD, CD19^−^ normal B cells can confound analysis. CD19^−^ normal B-cell precursors are present in bone marrow and peripheral blood (77–80) and can expand after CD19-targeted therapies (78, 80), complicating discrimination between malignant CD19^−^ blasts and normal cells. Flow cytometry can also generate non-specific signals, leading to false-positive or false-negative events. Thus, great care is needed when quantifying CD19^−^ cells.

Together, these findings support pre-existing CD19^−^ B-cell clones as a key driver of CD19^−^ relapse, while highlighting that normal CD19^−^ B cells and technical limits of current assays hinder precise pre-treatment assessment of these malignant clones.

### 2.2 Relapse due to CD19 downregulation

Beyond pre-existing CD19^−^ B-cell clones, another major mechanism of CD19^−^ relapse is loss of CD19 during CAR-T cell therapy. As with other targeted cancer therapies, selective pressure can drive cancer cells to gradually lose expression of the targeted antigen. For example, after haploidentical hematopoietic stem cell transplantation (HSCT), leukemia cells may lose mismatched HLA through acquired uniparental disomy of chromosome 6p, thereby escaping donor T cells and causing relapse (81). In CAR-T cell therapy, despite some conflicting data (40), higher antigen expression on cancer cells may correlate with better CAR-T cell function (82) and improved clinical outcomes (83), highlighting the need to understand antigen downregulation. In CD19 CAR-T cell therapy, CD19^−^ relapse due to CD19 downregulation is common. Key mechanisms include direct mutations, aberrant RNA splicing, and transcriptional/post-transcriptional dysregulation.

#### 2.2.1 CD19 downregulation due to direct mutation and aberrant splicing

Direct genetic mutations and aberrant RNA splicing are closely related central mechanisms of CD19 downregulation (71) and common routes by which CD19^+^ cancer cells escape CD19 CAR-T cells (84, 85). Under sustained CAR-T cell pressure, cancer cells can acquire *CD19* mutations and ultimately lose CD19 expression, leading to CD19^−^ relapse. The main CD19 alterations observed are described below.

##### 2.2.1.1 Intron retention

Black et al. (86) found that genes encoding splicing factors are highly mutated and that mRNAs are aberrantly spliced in B-ALL compared with normal pro-B cells. Thus, aberrant splicing is a hallmark of leukemia and can disrupt CD19 processing. Indeed, intron retention in CD19 produces a truncated protein lacking a functional transmembrane domain and is a common mechanism of resistance to CD19 CAR-T cell therapy in lymphoma and B-ALL (73, 84, 87–91).

In CD19 CAR-T cell therapy, the widely used CD19-specific single-chain variable fragments (scFvs), FMC63 (92, 93), recognizes an epitope in the extracellular domain encoded mainly by exon 4 (84, 94) later mapped more precisely to a region spanning exons 3 and 4 (95). Point mutations or abnormal splicing that cause intron retention can generate truncated CD19 isoforms that lack scFv binding sites and/or have a nonfunctional transmembrane domain. For example, Orlando et al. (84) described a splice acceptor site mutation that led to retention of intron 5 and destabilization of the transmembrane domain.

Among all *CD19* introns, intron 2 is most enriched in CD19^-^ relapses after CD19 CAR-T cell treatment (87–91, 96). Because intron 2 contains a premature stop codon (90), its retention eliminates the FMC63 binding site encoded by exon 4. In cell models, intron 2-retaining transcripts represent over 33% of all *CD19* mRNA (90), rising to 82% in B-ALL samples from patients who relapse after CD19 CAR-T therapy (88). Even before treatment, intron 2 retention accounts for 63% of all *CD19* isoforms, making it the dominant form in nearly all patients (88). These findings indicate that *CD19* is frequently mis-spliced in cancer cells, in line with the broader role of aberrant splicing in leukemia. PacBio long-read sequencing, however, suggests that in most patients with primary nonresponse or primary sensitivity to CD19 CAR-T cells, more than 70% of sequenced *CD19* transcripts are wild-type (97), though this does not exclude additional isoforms. Consistently, Li et al. (96) identified six alternatively spliced *CD19* mRNA isoforms, including the intron 2 retention variant, in Nalm-6 cells and B-ALL patient samples.

Multiple point mutations and RNA-binding proteins (RBPs) modulate splicing of *CD19* intron 2 (87, 88). Ziegler et al. (87) showed that Polypyrimidine tract binding protein 1 (PTBP1) is required for proper intron 2 excision. At diagnosis, 35% of B-ALL samples already carry a 2-nucleotide deletion in the PTBP1 binding site within intron 2 (87), predisposing to intron 2 retention and CD19^−^ relapse after CD19 CAR-T cell treatment. Domizi et al. (98) further reported that low expression of the transcription factor IKAROS in pro-B-like tumor cells correlates with CD19^−^ relapse in CD19 CAR-T cell-treated leukemia patients. IKAROS loss promotes intron 10 retention and further increases the already high frequency of intron 2 retention in B-ALL.

Overall, these data show that multiple CD19 isoforms—particularly the intron 2 retention variant—are already present in B-cell malignancies before CD19 CAR-T cell treatment. Pre-existing genetic alterations and dysregulated RBPs favor intron 2 retention. After CAR-T cell infusion, selective pressure may promote additional point mutations in *CD19* and expand newly mutated isoforms with a survival advantage. Ultimately, the intron 2 retention isoform can dominate, causing functional loss of CD19 and enabling tumor cells to evade CAR-T cell-mediated killing (**Figure 1**).

##### 2.2.1.2 Exon 2 deletion

Beyond intron 2 retention, another major mis-spliced *CD19* isoform in relapsed B-ALL after CAR-T therapy is exon 2 deletion (90, 97, 99). Exon 2 is required for proper CD19 folding; its loss, or in-frame mutations within this exon, causes misfolding and endoplasmic reticulum retention (100). As with intron 2 retention, RBPs are crucial for *CD19* exon 2 inclusion. Sotillo et al. (99) identified splicing factor Serine/Arginine-rich splicing factor 3 (SRSF3) as a key regulator of exon 2 retention, and SRSF3 is downregulated in CD19^−^ relapsed B-ALL (99), which may explain the enrichment of exon 2 skipping. Although frameshift mutations can introduce a premature stop codon and result in (partial) exon 2 loss (99–101), Sotillo et al. (99) suggested that exon 2 alternative splicing can bypass canonical frameshift and missense mutations, underscoring the central role of aberrant splicing in *CD19* mRNA processing.

The exon 2-skipped isoform, like that with intron 2 retention, is already present before CAR-T therapy (97, 101), implying clonal evolution under selective pressure. Truncated CD19 generated by exon 2 skipping can partially compensate for CD19 loss while escaping FMC63-based CD19 CAR-T cell recognition (99), increasing the chance that exon 2-skipped isoforms become dominant during therapy.

Given the extensive splicing abnormalities in B cell malignancies, cancer cells likely carry a wide range of *CD19* isoforms arising from mis-splicing or mutations, with rare isoforms present as subclones before treatment (88). Because wild-type CD19 is essential for B cell survival (102), untreated lymphoma and leukemia cells mainly express wild-type CD19. Under pressure from CD19-directed CAR-T cells, wild-type CD19 is lost and novel isoforms emerge, leading to “loss of CD19”. Cells with exon 2 skipping, and possibly intron 2 retention, appear to gain survival advantages and thus become the most common isoforms in CD19^−^ relapse after CAR-T cell treatment. Under alternative selective pressures, such as different treatment regimens or CAR-T constructs targeting distinct CD19 domains, other mis-spliced CD19 isoforms could instead dominate and drive CD19^−^ relapse (**Figure 1**).

Long-read sequencing has greatly improved detection of *CD19* splicing isoforms (97, 103) by enabling direct analysis of full-length transcripts. Schulz et al. (103) verified exon 2 skipping and intron 2 retention in *CD19* using long-read sequencing, but they did not observe the isoform with partially deleted exon 2, which is detected by cDNA-seq and reported in some studies (96, 99, 101). They proposed that a specific exon 2 RNA secondary structure may cause PCR polymerase slippage, generating a pseudo-truncated product and a false-positive cDNA-seq signal (103). Similar artifacts could affect other purported novel *CD19* splicing isoforms, highlighting the need to validate CD19 isoforms using direct long-read sequencing.

##### 2.2.1.3 Other potential mutations

In addition to aberrant splicing, coding mutations in CD19 also contribute to CD19 loss. Using an *in vitro* co-culture model of CD19^+^ Nalm-6 cells under CD19 CAR-T cell pressure, Li et al. (96) found that all splicing isoforms, including intron 2 retention and exon 2 skipping, together represented less than 50% of CD19 alleles in the relapsed CD19^−^ Nalm-6 cells, implying additional mechanisms. Several studies have reported numerous frameshift and missense CD19 mutations during CD19^−^ relapse (84, 88, 91, 99). These coding mutations may be at least partly drive CD19 loss, although it remains debated whether aberrant splicing or somatic mutation is the major cause.

To clarify the role of *CD19* mutations in CD19^−^ relapse, Chen et al. (104) performed deep whole-exome sequencing (WES) in B-ALL patients after CAR-T cell treatment, comparing CD19^−^ relapse with CD19^+^ relapse and pre-treatment samples. WES detects minor *CD19* variants with allele frequencies as low as 0.8%. In all 7 eligible CD19^−^ relapse cases, relapse-specific (absent at baseline) nonsynonymous *CD19* mutation variants with over 45% allele frequencies were identified, mainly non-frameshift insertions, frameshift deletions, and premature stop codons. In contrast, in 5 CD19^+^ relapse cases, no nonsynonymous *CD19* variants exceeded a 3% allele frequency (104). Only CD19, and not other B-cell markers, carried such relapse-specific mutations, indicating that they likely arise under CAR-T cell-mediated selective pressure.

Although pre-existing *CD19* mutation may initiate CD19^−^ relapse in some patients, Chen et al. (104) concluded that baseline mutation burden and genomic instability do not predict the CD19^−^ relapse after CAR-T cell therapy. They also found no clear association between CD19 mutations and poor clinical outcomes, as most patients with CD19 mutations still achieved remission, albeit in a small cohort (104). Conversely, a retrospective study showed that even the single-nucleotide polymorphisms (SNPs) at amino acid 174 affect response: the germline leucine 174 variant was linked to better outcomes than valine 174 in DLBCL patients with FMC63-based CD19 CAR-T cells (105).

CD19 CAR-T activity depends on recognition of a specific CD19 epitope. Alterations at this site can confer resistance without fully abolishing CD19 expression. Zhang et al. (106) reported that lymphoma patients with CD19 exon 3 alterations, including R163L and L174V, were resistant to FMC63-based CD19 CAR-T cells; whereas 21D4-based CD19 CAR-T cells effectively killed cells carrying these mutations and retained activity even in the absence of exon 1, 2, or 3 (106). Epitope mapping showed that FMC63 recognizes spatially adjacent loops encoded by exons 3 and 4 (95) and L174V likely induces a subtle conformational change that reduces FMC63 binding affinity (105). Among other scFvs, 3B10 targets a linear 12-amino acid stretch including the same exon 3 loop but not the exon 4 loop; whereas 4G7 overlaps with FMC63/3B10 at residues W140-G150 (exon 3 loop) and P200-P203 (exon 4 loop) (105). The 21D4 epitope remains undefined.

Different CD19 CAR-T products recognize different CD19 epitopes and impose distinct evolutionary pressures. 21D4-based CD19 CAR-T cells, for example, can clear CD19 isoforms that escape FMC63-based CAR-T cells (106). Even FMC63, 4G7, and 3B10—despite overlapping epitopes—show different tolerance to specific mutations (105), supporting the idea that each CAR design drives a unique pattern of CD19 isoform evolution. He et al. (92) solved cryo-EM structures of CD19 bound by scFvs FMC63 and SJ25C1, and used them to engineer scFvs with tuned tumor recognition sensitivity. Such structure-guided design may help anticipate CD19 alteration and guide the selection of CAR constructs optimized for specific CD19 isoforms. Costimulatory domains also shape selective pressure. In an unpublished study, Krawczyk et al. (91) showed that CD19-4-1BB-CAR-T cells preferentially eliminate CD19^high^ tumor cells, enriching for CD19^low^ cells. CD19 mutations then accumulate, ultimately yielding CD19^−^ clones lacking the target epitope. In contrast, CD19-CD28-CAR-T cells kill both CD19^high^ and CD19^low^ tumor cells, and no *CD19* mutations and splicing alterations were detected, indicating preserved epitopes. A similar pattern for CD19-CD28- versus CD19-4-1BB-based CAR-T cells was reported by Majzner et al. (107). Although CD19 may be downregulated after CD19-CD28-CAR-T cell treatment, this appears to result from transcriptional or posttranscriptional regulation rather than genetic alteration (91).

#### 2.2.2 Additional transcriptional and posttranscriptional changes in CD19 regulation

Beyond direct mutations and aberrant splicing, transcriptional, posttranscriptional, and epigenetic adaptations also contribute to CD19^-^ relapse in the absence of detectable genomic alterations. Duell et al. (108) did not find CD19 mutations or aberrant splicingin DLBCL patients with CD19^-^ relapse after CD19 CAR-T cell treatment, indicating that non-mutational adaptations of CD19^+^ cancer cells under CD19 CAR-T cell-mediated selective pressure may drive CD19^−^ relapse.

##### 2.2.2.1 Epigenetic and transcriptional regulation

**Figure 2:**
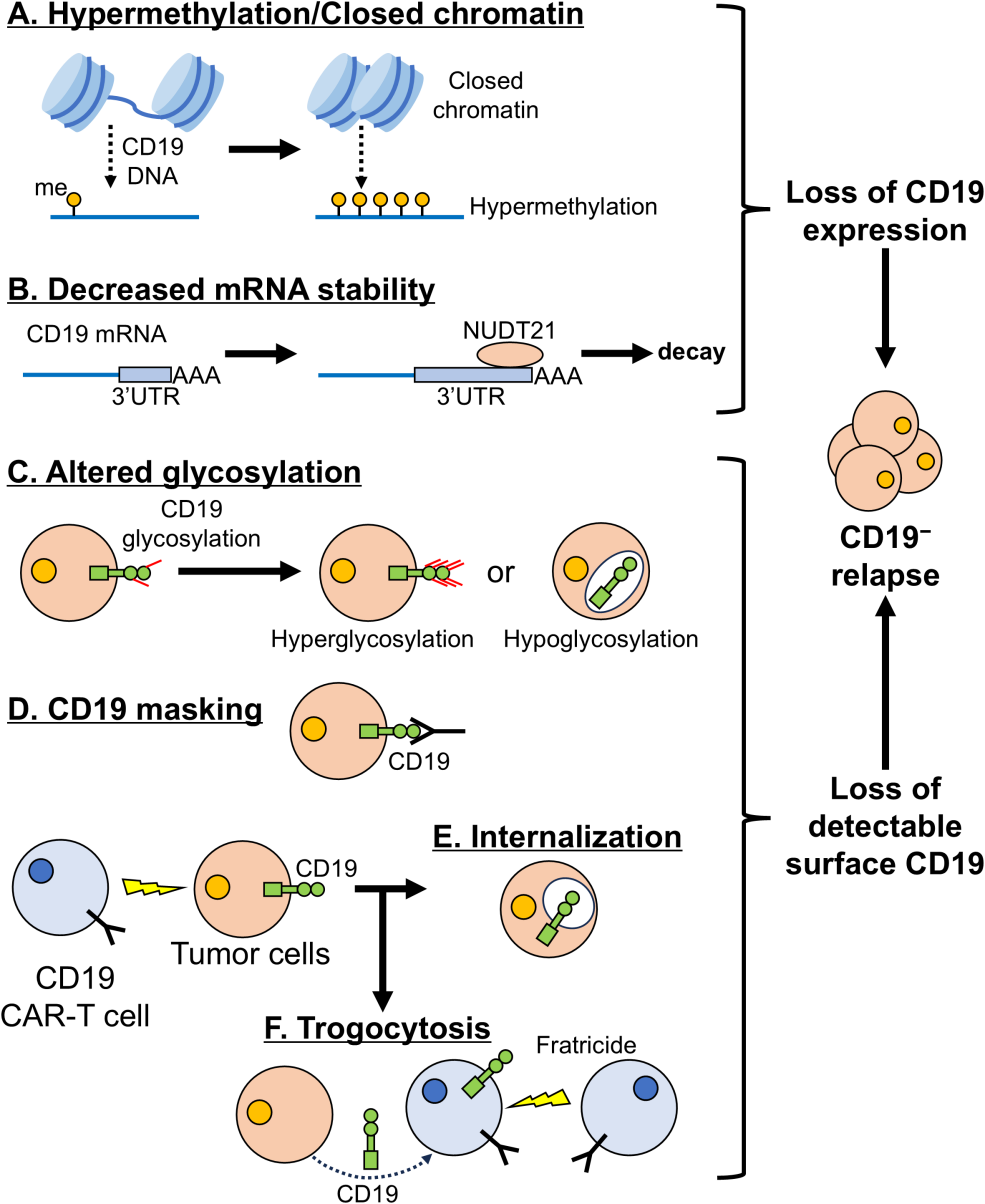
**Other mechanisms leading to loss of CD19.** Under selective pressures from CD19 CAR-T cells, CD19^+^ tumor cells can downregulate CD19 through either directly losing CD19 expression **(A, B)** or losing detectable surface CD19 **(C-F)**. **(A)** Reduced chromatin accessibility and CD19 promoter hypermethylation suppress transcriptional expression of CD19. me: DNA methylation. **(B)** Increased NUDT21 expression favors the use of distal polyadenylation site usage in CD19 mRNA, extending the CD19 3’UTR and destabilizing the transcript. **(C)** Altered glycosylation (hyper- or hypoglycosylation) either masks CD19 from CAR-T cell recognition or retains the protein in the Golgi apparatus. **(D)** Transient epitope masking occurs when proteins or antibodies bind surface CD19 and block CAR-T cell recognition. **(E)** After contact with CAR-T cells, tumor cells can internalize surface CD19, becoming transiently CD19^−^. **(F)** Tumor cells can transfer CD19 to CAR-T cells via trogocytosis, generating CD19^−^ tumor cells and CD19^+^ CAR-T cells that undergo T-cell fratricide. Together, these mechanisms drive CD19^-^ relapse. .

Epigenetic modifications and transcriptional dysregulation are important non-mutational mechanisms regulating CD19 expression during CAR-T therapy. To investigate tumor-intrinsic drivers of CD19 loss, Ledererova et al. (109) created an *in vivo* chronic lymphocytic leukemia (CLL) model of CD19^−^ relapse after CD19 CAR-T cell treatment. They showed that transient CD19^−^ escape is associated with CD19 promoter methylation, which can be partially reversed by the demethylating agent 5-aza-2’-deoxycytidine. Using single-cell RNA sequencing (scRNA-seq), Aminov et al. (110) identified a transcriptionally distinct CD19^low^ subpopulation enriched among resistant cells after CD19-directed therapy. Assay for transposase-accessible chromatin with sequencing (ATAC-Seq) revealed reduced chromatin accessibility at CD19 promoter regions in these cells, consistent with decreased CD19 expression. In addition, expression of CD19 activators CTNNBL1 and ​​CD81 is reduced, whereas expressions of CD19 inhibitor SOX4 is increased. In the absence of CD19 signaling, B-cell receptor (BCR) signaling alone can sustain proliferation of these resistant tumor cells (110) (**Figure 2A**).

##### 2.2.2.2 Regulation of CD19 RNA stability

Posttranscriptional regulation of CD19 mRNA stability is an important determinant of CD19 expression. A genome-wide CRISPR/Cas9 screen by Witkowski et al. (111) identified the RBP NUDT21 as a negative regulator of CD19. NUDT21 binds the *CD19* 3’ untranslated region (3’UTR) region and promotes distal polyadenylation site usage, producing long 3’UTR transcripts that are more prone to decay in transformed B-cell progenitors. NUDT21 loss shortens the 3’ UTR, stabilizes CD19 mRNA, and increases CD19 expression. In B-cell progenitor ALL (BCP-ALL) patients treated with CD19 CAR-T cells, higher NUDT21 mRNA levels correlate with CD19 loss at relapse (111) (**Figure 2B**).

##### 2.2.2.3 Altered CD19 glycosylation and trafficking

Posttranslational changes and trafficking defects can also reduce CD19 surface expression and drive CAR-T resistance. Glycosylation, the addition of glycans to proteins, is common on membrane proteins and affects CAR recognition; different scFvs vary in their sensitivity to antigen glycosylation (112). Thus, altered CD19 glycosylation can impair CAR binding and lead to pseudo-CD19^−^ relapse. In a CRISPR/Cas9 screen, Heard et al. (113) identified the Golgi intramembrane protease Signal peptide peptidase-like 3 (SPPL3) as a regulator of CD19 glycosylation and CD19 CAR-T cell resistance in ALL. Loss of SPPL3 causes CD19 hyperglycosylation, particularly at asparagine residue near the CAR epitope, directly mediating resistance. SPPL3 overexpression has the opposite effect, inducing CD19 hypoglycosylation and loss of CD19, which also confers resistance (113). Clinically, resistant ALL samples from CAR-T-treated patients harbor CD19 mutations with deletion of Y260 (CD19ΔY260) (114). CD19ΔY260 is hypoglycosylated and retained in the Golgi (**Figure 2C**).

Similar CD19^−^ relapse mechanisms have been seen with blinatumomab. In one early-relapse ALL case with wild-type CD19, the CD19 protein was hypoglycosylated and failed to reach the cell surface because of loss of CD81, a chaperone required for CD19 maturation and membrane trafficking (115). However, no CD81 mutation was found, and the cause of CD81 loss is unknown.

##### 2.2.2.4 CD19 loss via internalization and trogocytosis

Rapid CD19 internalization provides a non-genomic, early mechanism of CD19 downregulation after CAR-T engagement. Im et al. (116) showed that B-ALL cells decrease CD19 expression within hours of contact with CD19 CAR-T cells. CAR-T cells induce CD19 clustering at the immune synapse, followed by internalization. Later, CD19 expression in resistant cells is further reduced by transcriptional mechanisms (116). scRNA-seq and single-cell ATAC-seq indicated that pathways associated with B-cell activation and germinal center responses help maintain resistant CD19^low^ cells, and this resistance can be reversed with the BTK inhibitor ibrutinib (116). Blanco et al. (117) similarly observed rapid CD19 internalization and subsequent lysosomal degradation in leukemia cells after CD19 CAR-T cell treatment (**Figure 2E**).

Beyond intracellular retention, CD19 downmodulation can also occur through trogocytosis, in which CD19 is transferred from leukemia cells to CAR-T cells (118). Interaction with either CD19-CD28- or CD19-4-1BB-CAR-T cells results in loss of CD19 from ALL cells and acquisition by CAR-T cells, yielding CD19^−^ tumor cells and CD19^+^ T cells. This promotes tumor escape and T cell fratricide, and surviving CAR-T cells become prone to exhaustion (**Figure 2F**). Because CD19-CD28-CAR-T cells more effectively target cells with low antigen levels, fratricide is more pronounced with this construct. However, their high sensitivity to CD19^low^ cells also allows a second infusion of CD19-CD28-CAR-T cells to rescues relapsed ALL after initial therapy (118).

##### 2.2.2.5 Pseudo-CD19^−^ relapse due to CD19 masking

Beyond changes that alter the surface expression of wild-type CD19, membrane CD19 can be masked, making cells invisible to CAR-T cells. Fitzgerald et al. (119) reported two patients with aggressive mature B-cell lymphoma treated with tafasitamab, an anti-CD19 antibody, in whom a low antibody-CD19 dissociation rate led to transient masking of CD19 on lymphoma cells. This masking rendered cells undetectable to CD19 CAR-T cells and delayed subsequent CAR-T cell treatment. Ruella et al. (120) described another form of CD19 masking during CAR-T cell manufacturing: a single leukemia B cell was inadvertently transduced with the CAR gene. Although this cell expressed wild-type CD19, the CAR produced by the same cell bound to its own surface CD19, masking the antigen and ultimately causing a CD19^−^ relapse (**Figure 2D**).

Overall, CD19^-^ relapse after CAR-T therapy arises from multiple interconnected mechanisms, including genetic alterations (mutations and aberrant splicing), epigenetic/transcriptional dysregulation, posttranslational changes (e.g., glycosylation), dynamic cellular processes (internalization and trogocytosis), and epitope masking. These mechanisms are largely driven by CAR-T-mediated selective pressure, allowing pre-existing subclones or *de novo* adaptations to escape immune surveillance.

### 2.3 Relapse due to lineage switch

Lineage switching is another mechanism by which CD19^+^ leukemia or lymphoma cells evade CD19 CAR-T cells. It involves complete or partial conversion from a B-lymphoid to a myeloid phenotype, resulting in CD19^−^ relapse. This phenomenon is rare in B-ALL patients treated with chemotherapy (less than 1%) (121–123) but increases to about 8% among relapsed patients receiving CD19 CAR-T cells or other CD19-directed immunotherapies (124, 125). Lineage switch from lymphoma to acute myeloid leukemia (AML) has also been described (126). An international analysis from Project EVOLVE examined lineage switching after immunotherapy, including CAR-T cells (127). Among 70 cases of lineage switch from B-ALL to AML, mixed phenotypic acute leukemia, or acute leukemias of ambiguous lineage, 81.4% occurred within 6 months of immunotherapy, and 64.3% harbored KMT2A rearrangements. Prognosis was poor, with remission rates under 40% and a median OS of 4.8 months. Thus, early recognition of lineage switch—as opposed to misclassification as a second malignancy—and investigation of its mechanisms are critical for optimal management. Evidence on lineage switching after CAR-T cell therapy largely comes from case reports and small cohorts, reflecting both its rarity and the challenges of modeling it *in vitro* or *in vivo*. Key case reports of lineage switch relapse after CD19 CAR-T cells, and the methods used to establish clonal relatedness between relapse and original blasts are summarized in **Table 2** and discussed further in Section 3.1. Project EVOLVE has contributed additional, partly overlapping cases (127). Across these reports, lineage-switch relapses exhibit highly heterogeneous immunophenotypes, complicating efforts to fully characterize this process. In this section, we review current data on lineage switch after CAR-T cell therapy and outline proposed mechanisms.

**Table 2:** Immunophenotype of lineage switch after CD19 CAR-T cell therapy in case reports.

Study/age	Diagnosis pre-CAR-T	Immunophenotype pre-CAR-T	Relapse diagnosis (time after CAR-T)	Relapse immunophenotype	How to access the clonal relationship	Rearrangement/ fusion gene	Salvage treatment
(128) 13mo	r/r B-ALL (CD19^+^ relapse after the second CAR-T)	+: CD19^dim/mod^, CD22^bri^, CD10^−/dim^, TdT, CD34, CD38, CD58, HLA-DR^bri^, CD123	AML (10mo)	+: CD13, CD33^P/dim^, CD34, CD117, CD123, CD11b^P^, CD38^mod^, CD7 (but Pax5^weak^ by IHC)	FISH demonstrated persistent loss of the ETV6 (12p13)	ZNF384 (TCF3::ZNF384)	Palliative care, died 4mo later
		−: CD24, CD13, CD33, MPO		−: CD19, CD10, CD20, CD24, MPO, TdT, and CD22			
(129) 52yo F	r/r B-ALL	+: CD45, CD19, CD22, CD38, HLA-DR, CD15, CD33^L^, CD13^dim^, TdT^dim^	AML (mono) (35d)	+: CD13^dim^, CD64, HLA-DR^dim^, CD15, CD33^H^, CD71, MPO	Karyotyping/FISH identified persistent MLLr; IGH deep sequencing identified persistent malignant clone; CGAT demonstrated clonal evolution	KMT2A	Reinduction followed by allo-HSCT, died 147 days after CD19 CAR-T-cell infusion due to relapsed AML
		−: CD64, CD14, CD4		−: CD19, CD20, CD22, CD24, CD79a^cyto^, CD34, CD14			
(129) 18yo F	r/r B-ALL	+: CD19, CD38, CD58, CD22^dim^, HLA-DR, CD34, CD45	CD19^−^ abnormal myeloblasts (30d after CAR-T);	CD4, CD56^sub^, CD64^int^, CD13, CD33^bri^, CD38, HLA-DR, CD34, CD45, CD117^sub^ and CD71	Karyotyping/FISH identified persistent MLLr; Myeloblasts showed no shared IGH rearrangement observed in B-ALL (assessed by IGH deep sequencing) ➔ relapse from immature stem cell clone	KMT2A	Failed HD reinduction chemotherapy, died
		−: CD4, CD10, CD13, significant CD33, CD56, CD64, CD117		−: No abnormal B cell CD19			
(130)	B-ALL	+: CD19, CD22, CD10, CD34^mod^, HLA-DR, CD33, CD11b^L^	CD19^+^ lymphoid- myeloid mixed phenotype	+: CD19, CD22, CD10, CD34^mod^, HLA-DR, CD33, CD11b^mod^	Not reported	Normal karyotype	Not reported
		−: CD14, CD117		−: CD14, CD117			
(130)	B-ALL	+: CD19, CD22, CD34, HLA-DR, CD33^mod^	CD19^−^ myeloid phenotype	+: CD34, HLA-DR, CD33, CD11b, CD117^L^	Not reported	KMT2A	Not reported
		−: CD10, CD14, CD117, CD11b^L^		−: CD19, CD22, CD10, CD14			
(131) 5yo M	r/r B-ALL	+: CD2, CD7, CD10, CD13, CD19, CD22, CD34, CD56^P^, CD79a, CD123, HLA-DR, TdT	Myeloid tumors, (3yr)	+: CD4, CD7, CD11b, CD13, CD33, CD38, CD56, CD58, CD64, CD71^dim^, CD117, CD123^P^, HLA-DR, CD43, CD45, CD68, CD163^L^, Lysozyme	Persistent fusion and mutations detected by cytogenetics and NGS	KMT2A	HD chemotherapy followed by HSCT, died from TRM
(132) 3yo F	r/r precursor-B-ALL	Not reported	AML (7mo)	Not reported	WGS of serial samples showed shared mutations and variations ➔ AML as the direct descendant of the ALL	No common cytogenetic aberrations	HD chemotherapy, lives in CR
(133) 14yo F	r/r B-ALL (relapse from the initial CD22 CAR-T)	+: CD22, CD19, CD10, CD33^P/dim^, CD38, CD45^dim^, CD58, CD71	Acute leukemia of ambiguous lineage: T/myeloid (1yr after consol-HSCT)	+: CD3, CD33, CD34, CD117, CD133, MPO^sub^	Persistent ETV6::RUNX1 rearrangement	ETV6:: RUNX1 fusion	Multiple lines of therapy, only Venetoclax + ARA-C achieved short-term CR, patients died from LS 10mo later
		−: CD2, CD13, CD14, CD20, CD34		−: CD19, CD22, CD5, CD11c, CD14, CD13, CD123			
(124) 0.3yo M	B-ALL	+: CD19, CD22, CD34, CD38, CD58, HLA-DR, surface lambda	AML (mono) (8.5mo)	+: CD7^sub^, CD33, CD36, CD64, HLA-DR, MPO	Cytogenetic and molecular studies and NGS showed shared genetic alterations with additional mutations	KMT2A	Gemtuzumab with Fludarabine and HD Cytarabine, followed by HSCT, lives in CR
		−: CD10, CD13, CD15, CD20, CD33, CD56, MPO, TdT, surface Kappa		−: CD13, CD10, CD19, CD20, CD22, CD79a^cyto^, CD2, CD3, CD4			
(134) 46yo F	r/r Ph+ B-ALL	+: CD34, CD10, CD19, CD20, CD22, CD38, HLA-DR	AML (3yr)	+: CD34, CD13, CD33, CD38, CD117, CD15, HLA-DR	Not reported	No common aberrations (negative BCR::ABL1 at relapse) FLT3::ITD mutation + PAX5 SNP by NGS	DCAG, lives with CR
(135) 27yo M	r/r B-ALL	+: CD10, CD79a^cyto^, CD34, CD19, CD22, CD28, TdT^cyto^, CD58	AML (5wk)	+: CD38^H^, CD33^H^, CD36^H^, HLA-DR^H^, CD13, CD123, TdT, CD34^P^, CD11b^P^, CD11c^P^, CD64^P^, CD15^w^, MPO^w^	Persistent FLT3::ITD and BCORL1 mutations by targeted sequencing; scDNA for clone structure inference; scRNA for cell developmental trajectory analysis	No other typical fusion genes	Myeloid chemotherapy (azacitidine, cytarabine, venetoclax, and ibrutinib), remains in remission
				−: CD117, CD14, CD79a^cyto^, CD19, CD22, CD20			
(135) 26yo M	r/r B-ALL	+: HLA-DR, CD19, CD22, CD10, CD38, CD58	AML (1mo)	+: CD34^H^, CD13^H^, CD33^H^, CD123^H^, CD36^P^, CD38^w^, CD11b^w^, CD4^w^	Clonal evolution pattern by WES and targeted sequencing; BCR sequencing showed AML had identical Ig sequence as ALL ➔ AML from direct myeloid reprogramming process; scRNA for cell developmental trajectory analysis ➔ pro-B-like blast as the initiation of reprogramming	EP300:: ZNF384 fusion	Resistant to salvage treatment, died 3mo after CAR-T
				−: CD117, HLA-DR, MPO, TdT, CD14, and other myeloid and lymphoid markers			
(136) 56yo F	r/r Ph^+^ B-ALL	+: CD34, CD10, CD19, CD22, CD79a^cyto^, CD45, CD117^dim^, CD81	AML (11mo)	+: CD34, HLA-DR, CD13, CD33, CD117, CD38, CD45^sub^	Persistent the BCR::ABL1 fusion, karyotype and IGH rearrangement upon AML relapse; NGS revealed persistence of the mutations	BCR::ABL1 multilineage	Reinduction of dasatinib and venetoclax, maintained by dasatinib, patient lives in CR
		−: HLA-DR, MPO, CD7, CD14, CD2, CD15, CD11b, CD64, CD56, CD38, CD123, CD13, CD33, CD20, CD3, CD4, CD8,		−: CD7, CD10, CD20, CD19, CD14, CD2, CD15, CD11b, CD64, CD56, CD3, CD4, CD8			
(137) 21yo M	r/r B-ALL	+: HLA-DR, CD10, CD15, CD19, CD22, CD34, CD38, CD58, CD123, CD79a^cyto^, TdT, CD33^sub^ (Major population of lymphoblasts with a minor aberrant myeloblast subset)	Acute monocytic leukemia, from the minor myeloblast subset (19d)	+: CD13, CD33^H^ (details not reported)	scRNA and WES predicted clonal evolution: HSCs differentiated into GMPs and then lineage cells ➔ CD19 CAR-T cells accelerated myeloid evolution	No common fusion genes	Ineffective salvage chemotherapy, died

IHC, immunohistochemistry; AML, (mono): AML, with monocytic differentiation; NGS, next-generation sequencing; CGAT, chromosomal genomic array testing; MPO, myeloperoxidase; HD, high-dose; CR, complete remission; TRM, treatment related mortality in CR; WGS, whole genome sequencing; consol-HSCT, consolidative HSCT; ARA-C: ,ytarabine; WES, whole exon sequencing; SNP, single nucleotide polymorphism; DCAG, Decitabine, low-dose cytarabine, aclarubicin, and granulocyte colony-stimulating factor; scDNA, single-cell DNA sequencing; scRNA, single-cell RNA sequencing; HSC, hematopoietic stem cell; GMP, granulocyte macrophage progenitors; ^H,^ high; ^dim,^ diminished; ^int^: intermediate; ^P,^ partial; ^sub,^ subset; ^cyto,^ cytoplasmic; ^W,^ weak; ^L,^ low.

#### 2.3.1 KMT2A rearrangement as a major risk factor for lineage switch

Although mechanisms remain incompletely defined, KMT2A/Mixed Lineage Leukemia (MLL) rearrangement is the most consistently reported risk factor for lineage switch and subsequent CD19^−^ relapse after CAR-T cell treatment (124, 125, 129, 131, 138–141). In a retrospective cohort of 420 children and young adults with r/r B-ALL treated with CD19 CAR-T cells, 12 of 166 relapsed patients had a lineage switch, and KMT2A rearrangement (KMT2Ar) was the only pre-infusion factor associated with this outcome (125). Among all 420 patients, 38 had KMT2Ar, of whom 9 developed a lineage switch. Other studies similarly showed that at least 50% of lineage switch cases after CD19-directed immunotherapies carry KMT2Ar (142, 143), suggesting that KMT2Ar-related lineage switch may be a common response to immune-selective pressure.

**Figure 3:**
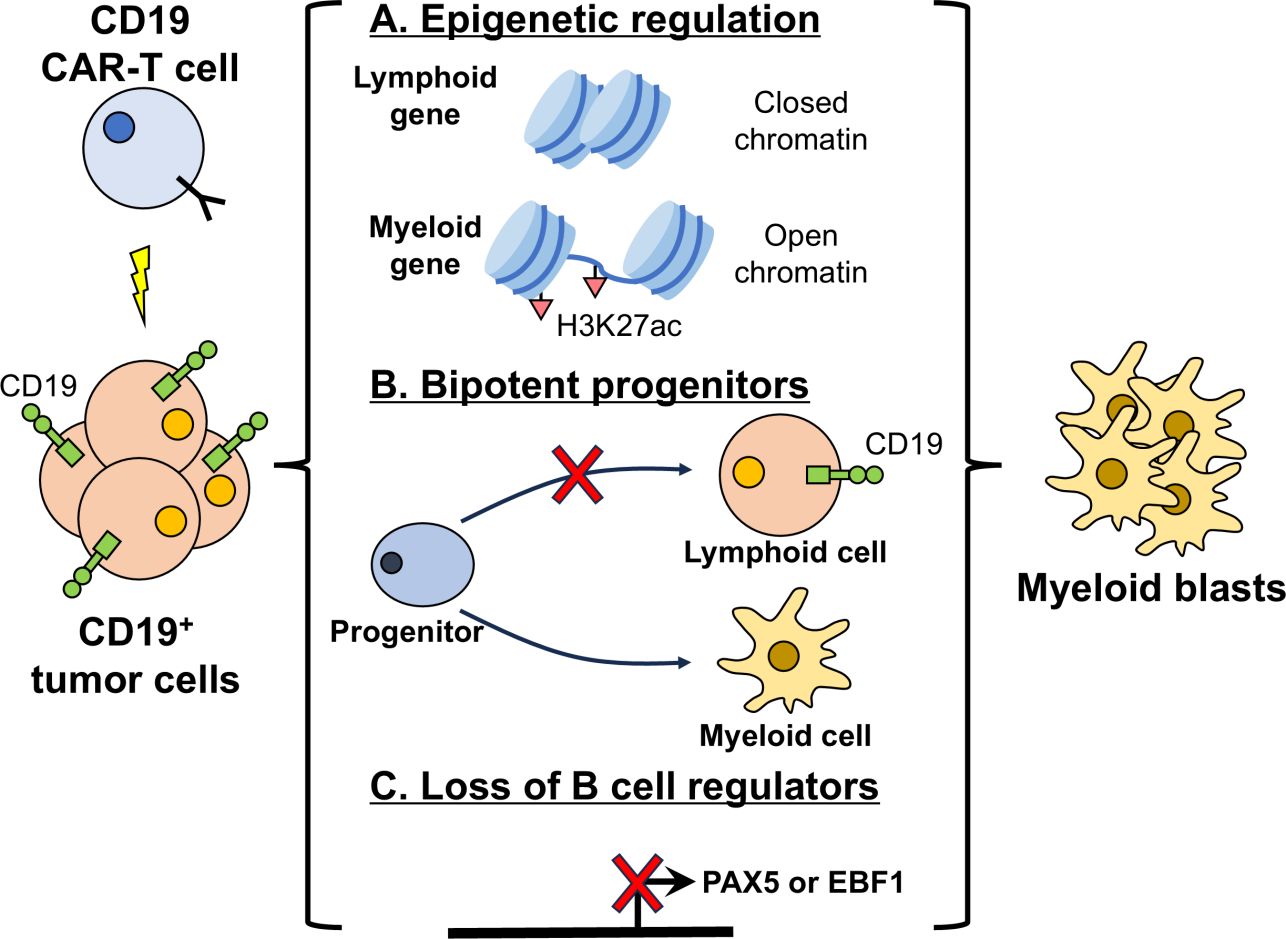
Lineage switch from lymphoid to myeloid phenotype as an escape mechanism from CD19 CAR-T cells. Lineage switch allows CD19^+^ lymphoid tumor cells to evade CAR-T cells by converting to CD19^-^ myeloid malignancies. Mechanisms include: **(A)** Epigenetic reprogramming, in which lymphoid genes are silenced by reduced chromatin accessibility, while myeloid genes are activated through increased chromatin accessibility and enhanced H3K27 acetylation. **(B)** Bipotent progenitors: biphenotypic lymphomyeloid progenitor cells exist prior to treatment. Under CAR-T-mediated selective pressures, these progenitors preferentially differentiate into the myeloid lineage. **(C)** Loss of B-cell regulators, where reduced expression of B-cell transcriptional factors PAX5 and EBF1 leads to loss of lymphoid identity and facilitates myeloid conversion.

​​ Despite this strong association, the biology underlying the KMT2Ar-driven lineage switch is unclear. One hypothesis is that therapy-resistant progenitor cells with myeloid potential give rise to the switched phenotype (129), although the connection between KMT2Ar and these progenitors is not fully resolved. Because KMT2Ar is linked to epigenetic dysfunction (144), epigenetic mechanisms likely contribute. In one ALL patient who switched lineage three years after CD19 CAR-T cell therapy, alterations in KMT2A and PHF6 suggested potential chromatin remodeling (131). Tirtakusuma et al. (145) proposed that epigenetic reprogramming underlies lineage switch in KMT2A::AFF1 ALL, a rearrangement common in infant ALL. The switch is associated with altered chromatin accessibility and transcriptional regulation and can be directly induced by dysregulation of chromatin modifiers such as Chromodomain helicase DNA-binding 4 (CHD4) (**Figure 3A**). In this model, the myeloid phenotype does not arise from a pre-existing myeloid subclone but emerges from various differentiation stages of ALL, from immature progenitors to committed B-cell precursors (145). Consistently, Jacoby et al. (130) failed to detect pre-existing myeloid subclones before CAR-T cell infusion. In contrast, Chen et al. (146) identified pre-existing lymphomyeloid progenitors and myeloid cells in KMT2Ar B-ALL that appear to underpin lineage switch after CAR-T cell therapy. These conflicting findings indicate that the mechanisms of lineage switch are likely heterogeneous and patient-specific.

#### 2.3.2 Lineage switch in other rearrangement

Beyond KMT2Ar-positive disease, lineage switch after CAR-T cell therapy also occurs in B-ALL without KMT2Ar, including Ph^+^ B-ALL (134), CRLF2-rearranged (Ph-like) B-ALL (147), and r/r B-ALL with normal-appearing karyotypes (148). It has likewise been reported in B-ALL with ZNF384 rearrangement (EP300::ZNF384 (135) and TCF3::ZNF384 (128)). Dickerson et al. (149) showed that hematopoietic stem and progenitor cells (HSPCs) overexpressing TCF3::ZNF384 or EP300::ZNF384 display aberrant H3K27 acetylation and enhancer activation, producing lineage-ambiguous leukemia with both B-cell and myeloid markers and a myeloid bias. These bipotent leukemia progenitors may underlie the tendency of CD19^+^ B-ALL to undergo lineage switch under CAR-T-mediated selective pressure. In a murine E2a:PBX1 ALL model, post-CAR-T cell lineage switch similarly correlates with loss of H3K27ac at B-cell markers and gain at myeloid enhancers (130) (**Figure 3A**).

#### 2.3.3 Bipotent progenitors and lineage switch

Pre-existing biphenotypic lymphomyeloid progenitors also promote lineage switch after CAR-T cell treatment. Multi-omics data from primary non-responders to CD19 CAR-T cells revealed pre-existing subclones co-expressing progenitor, lymphoid, and myeloid markers (97), indicating high lineage plasticity. Loss of IKAROS in B-ALL cells both disrupts normal CD19 splicing and increases expression of progenitor and myeloid markers (98). Saygin et al. (150) reported that 16% of adult ALL cases carry myeloid malignancy-related mutations that originate in HSPCs before ALL onset. These HSPCs expand to generate lymphoid malignant cells and “normal” myeloid cells (150), placing ALL in a pre-myeloid malignant state and predisposing it to lineage switch under CAR-T-induced selective pressure (**Figure 3B**).

#### 2.3.4 Alteration of B-cell transcriptional regulators and lineage switch

**Figure 4:**
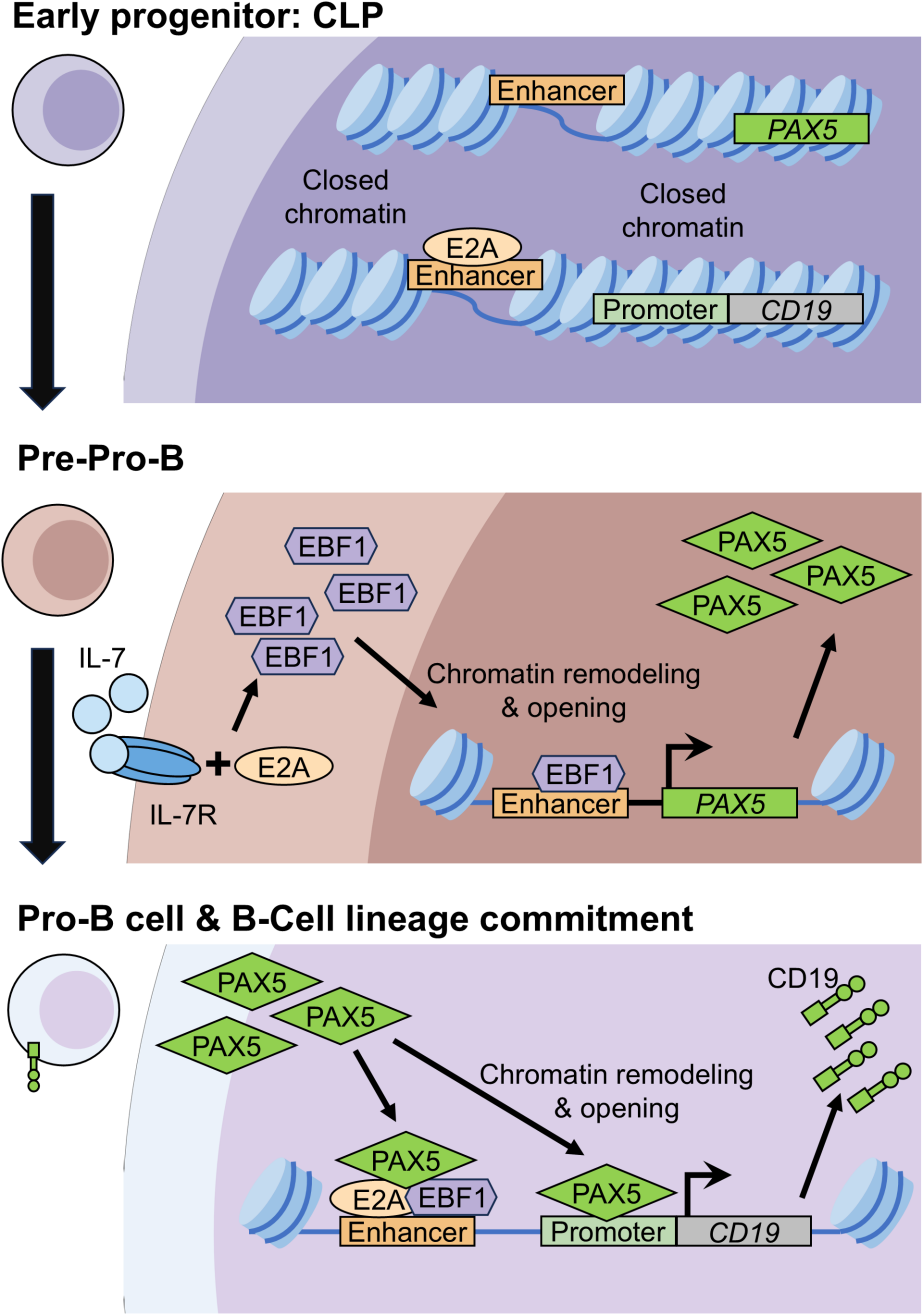
Regulation of CD19 expression. In multipotent common lymphoid progenitors (CLPs), the upstream CD19 enhancer is primed by chromatin remodeling and opening, accompanied by binding of E2A (E12/E47). At the same time, the PAX5 enhancer is also poised. B-cell lineage commitment in CLPs and transition to pre-pro-B cells are initiated by E2A together with IL-7/IL-7 receptor (IL-7R) signaling, which induce Early B-cell Factor 1 (EBF1). EBF1 then promotes chromatin remodeling and opens PAX5 promoter regions in pre-pro-B cells. In the presence of an already accessible enhancer, this results in PAX5 upregulation. Elevated EBF1 and PAX5 further reinforce B-cell lineage commitment and drive progression to the pro-B-cell stage. During this differentiation step, EBF1 binds the CD19 enhancer (already opened and occupied by E2A), followed by PAX5 recruitment. PAX5 also binds CD19 promoter regions, triggering chromatin opening, potentially involving CpG demethylation and histone modifications that favor transcriptional activation. Collectively, these events culminate in CD19 expression.

Paired Box 5 (PAX5) is a key regulator of B-cell identity (151) (**Figure 4**). A pre-existing KMT2A wild-type ALL subclone with hemizygous PAX5 loss was shown to be prone to lineage switch after CAR-T cell treatment (132), with treatment selecting for complete PAX5 loss and lineage conversion. In Ph^+^ B-ALL, PAX5 mutations are also detected and may underlie post-CAR-T lineage switch (134). In B-ALL treated with other therapies, PAX5 mutations are likewise strongly linked to lineage switch (152, 153). In murine ALL models, lineage-switched samples show little motif enrichment for Pax5 and another B-cell master regulator Early B-cell factor 1 (Ebf1) (130), and deletion of either gene reproduces lineage switch under CD19 CAR-T-mediated pressure (130), underscoring the central roles of PAX5 and EBF1 in lymphoid-myeloid conversion (**Figure 3C**).

Taken together, lineage switch is a distinct, high-risk mechanism of CD19^-^ relapse after CD19 CAR-T therapy. It is strongly associated with specific genetic backgrounds, particularly KMT2A rearrangement. Under CAR-T-driven selective pressure, lineage conversion is promoted by bipotent lymphomyeloid progenitors, epigenetic dysregulation that biases transcription toward myeloid programs, and disruption of key B-cell transcriptional regulators (PAX5, EBF1), which erodes lymphoid identity and enables lymphoid-myeloid transition.

## 3 Part II: clinical strategies for CD19^-^ relapse after CAR-T cell treatment

### 3.1 Diagnostic and treatment strategies for CD19^+/-^ relapse after CD19 CAR-T cell therapy

**Figure 5:**
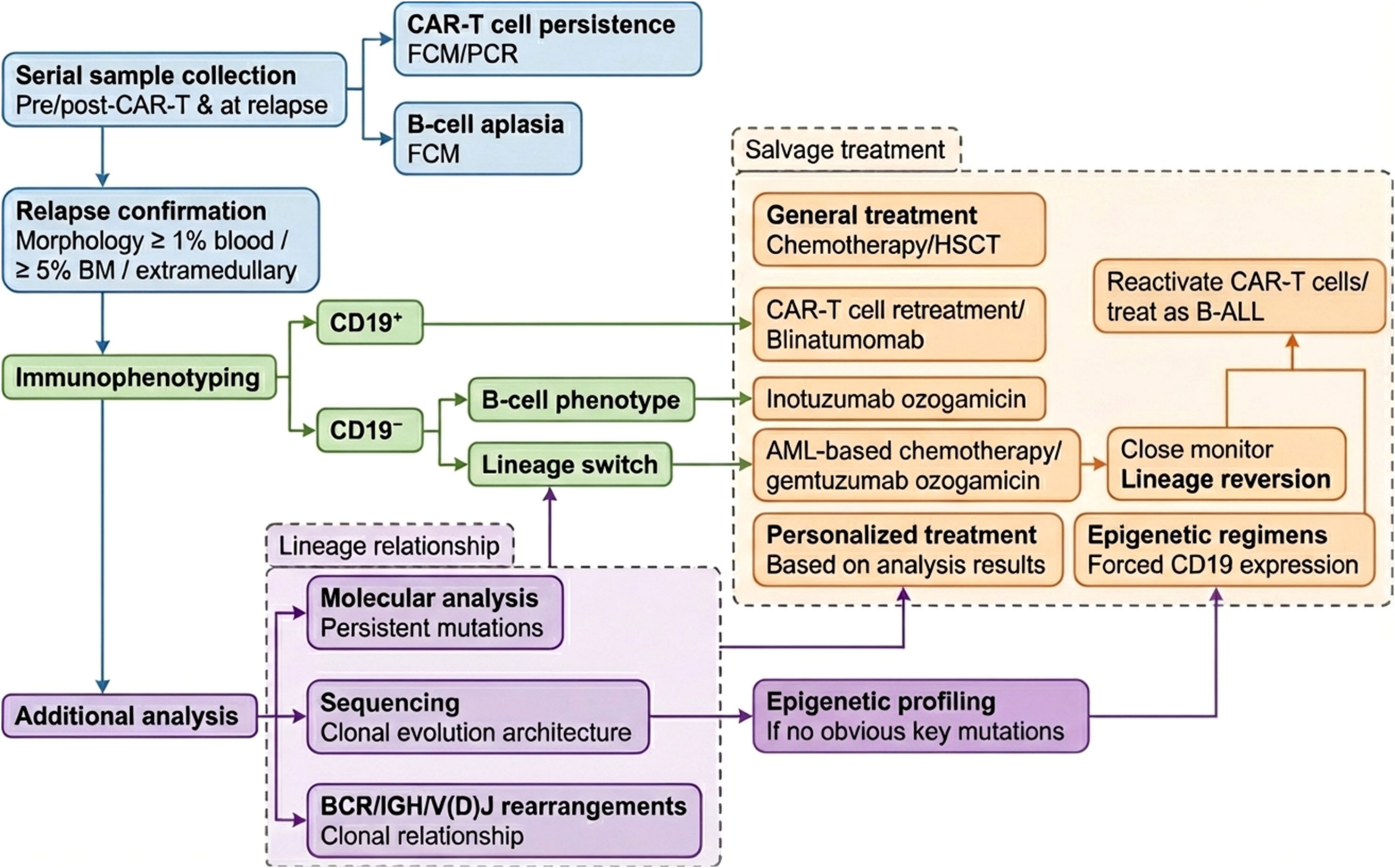
Workflow for diagnosis and salvage therapies for relapse after CD19 CAR-T cell treatment. FCM, flow cytometry.

Relapse after CD19 CAR-T cell therapy—whether CD19^+^ or CD19^-^—remains a major clinical challenge with poor prognosis (154). The mechanisms of CD19^-^ relapse are complex and not fully defined, and no standardized diagnostic criteria or treatment strategies are currently available. Based on our experience and a comprehensive literature review (including the studies summarized in **Table 1** and **2**) (127, 154), we propose a practical clinical workflow to guide management (**Figure 5**).

#### 3.1.1 Key monitoring indicators and risk factors for CD19^-^ relapse

This workflow emphasizes serial sampling and analysis at key time points: before CAR-T infusion, after CAR-T therapy, and at relapse. In parallel, two parameters should be monitored continuously: CAR-T cell persistence and B-cell aplasia (loss of normal B-cells). These can be assessed by flow cytometry. Additionally, RT-PCR for CAR transgenes, used in many studies (e.g (1).,), offers a highly sensitive method to track CAR-T kinetics. Notably, absolute CAR-T cell counts do not always correlate with antitumor activity, as functionally inactive but engrafted CAR-T cells have been reported (21). In contrast, depletion of CD19^+^ normal B-cells provides an indirect readout of functional CD19 CAR-T activity. Sustained B-cell aplasia is therefore widely used as a surrogate for ongoing CD19 CAR-T function (155). At relapse, persistence of functional CAR-T cells is strongly associated with CD19^-^ relapse, whereas absence of detectable CAR-T cells usually indicates CD19^+^ relapse (41). Thus, loss of B-cell aplasia together with impaired CAR-T persistence can help classify relapse type. Consolidative HSCT after CAR-T therapy benefits survival and reduces relapse risk (17, 18, 39, 41, 156) and is generally recommended when loss of B-cell aplasia is detected (39).

Prior exposure to blinatumomab is a key risk factor for CD19^-^ relapse. Evidence shows a strong association between prior blinatumomab therapy and subsequent CD19^-^ relapse (39, 47). Blinatumomab may downregulate CD19 expression on tumor cells, and patients with reduced CD19 expression after blinatumomab and before CAR-T therapy have the poorest outcomes and highest rates of CD19^-^ relapse (39). This likely reflects selection pressure on CD19 expression beginning with blinatumomab, rather than CD19 CAR-T therapy alone, underscoring the importance of prior treatment history when interpreting relapse mechanisms.

Additional potential risk factors for CD19^-^ relapse include high tumor burden (36, 47) and corticosteroid use for cytokine release syndrome (47). High tumor burden may indicate greater tumor heterogeneity and a higher probability of antigen-escape clones. And high tumor burden is often seen in heavily pretreated, highly refractory/relapsed disease in which tumors are already under strong selection pressure. Corticosteroids may further suppress CD19 CAR-T function and reduce their selective pressure, facilitating tumor survival and evolution under attenuated immune constraint.

#### 3.1.2 Criteria for CD19 status and relapse type

The risk factors described above help estimate relapse risk and evaluate relapse status indirectly, but direct analysis of relapse blasts is required to define the relapse type. Morphologic relapse is commonly defined as ≥1% blasts in peripheral blood, ≥5% blasts in bone marrow, or any extramedullary disease (1, 12, 13, 40, 42, 47). CD19 expression at relapse is assessed by flow cytometry and classified as follows (39):

1. CD19 intensity on normal mature B cells and isotype controls is used as the baseline.
2. CD19^+^: moderate to bright CD19 expression in >90% of blasts.
3. CD19^dim^: CD19^+^ blasts with intensity lower than normal mature B cells.
4. CD19^partial^: 50-90% CD19^+^ blasts in the leukemic population.
5. CD19^-^: >50% of blasts lacking CD19 expression in ALL, including bimodal populations where most blasts are CD19^-^.
6. Lineage switch: blasts with a surface immunophenotype consistent with myeloid markers (see next section for flow panels).

#### 3.1.3 Immunophenotypic assessment before CAR-T and at relapse

Relapse is first suspected based on morphology, but flow cytometry is the gold standard for defining its immunophenotype. As shown in **Table 2**, post-CAR-T relapse, especially lineage-switch relapse, is often immunophenotypically complex. Pre-CAR-T profiles are also heterogeneous because of extensive prior therapies, making panel selection challenging. Based on key reports (including (157), project EVOLVE (127) and the studies in **Table 1** and **2**), we suggest the following panels:

1) Preliminary panel: CD45; CD34; B-cell markers CD19, CD22; myeloid markers Myeloperoxidase (MPO), CD33, CD117; monocyte markers CD64, CD11c.

2) Extended panel:

- B-cell: CD19, CD20, CD22, cytoplasmic CD79a, CD10, CD24;
- Myeloid: MPO, CD33, CD117, CD13, CD11b, CD15, CD123;
- Monocyte: CD11c, CD14, CD64, lysozyme, CD36;
- Differentiation: CD34, CD38, HLA-DR, TdT;
- T-cell: CD3, CD4, CD8;
-Others/pan-markers: CD45, CD56;

Because CD19^-^ and lineage-switch relapses are highly heterogeneous, immunophenotypes must be interpreted individually. The Project EVOLVE classification with case examples (127) can aid clinical decision-making.

Beyond relapse assessment, thorough pre-CAR-T immunophenotyping using these panels is crucial. It can uncover minor pre-existing clones—such as CD19^-^ or myeloid-featured subsets—that may drive later relapse. For instance, a pre-existing minor CD33^+^CD19^weak^ subclone has been directly linked to rapid lineage-switch relapse after CAR-T treatment (137). Although detailed immunophenotyping is standard at diagnosis, it is often omitted immediately before CAR-T cell infusion. Before CAR-T cell therapy, most patients have undergone multiple lines of therapies, which may allow rare subclones to emerge. Repeating a comprehensive panel right before infusion can reveal these clones and support adaptation of treatment strategies.

RT-PCR is recommended to complement flow cytometry, and sequencing technologies discussed later can serve as alternatives or adjuncts. Compared with flow cytometry, RT-PCR and sequencing offer: higher sensitivity for weakly expressed genes (3); the ability to track changes in immunoglobulin gene rearrangements over time (45, 47, 83), shedding light on tumor evolution (37); detection of additional lymphoid and myeloid markers not covered by standard panels. For example, the B-cell lineage-limiting transcription factor PAX5 is a nuclear protein typically assessed by immunohistochemistry (IHC); its expression helps distinguish complete from partial lineage switch even when surface B-cell markers (CD19, CD20, CD22) are absent (128). However, IHC is not routinely used for liquid tumors such as leukemia.

#### 3.1.4 Clonal evolution assessment for primary and relapsed disease

Beyond immunophenotyping, genetic and molecular studies are central to defining the clonal relationship between primary disease and relapse. This is especially important in lineage-switch relapse, where clinicians must distinguish true relapse from a second neoplasm. Conventional tests—such as fluorescence *in situ* hybridization (FISH) and detection of characteristic cytogenetic abnormalities—are widely used (128, 129, 133). Persistence of fusion genes or specific rearrangements in both relapsed myeloid blasts and original lymphoid blasts supports a shared clonal origin, but these methods are often inadequate for detailed clonal analysis. High-throughput sequencing/technologies can more accurately define clonal relatedness by detecting shared mutations in primary and relapse samples. Common approaches include WES (135, 137), next-generation sequencing (NGS) (124, 131, 136), whole-genome sequencing (WGS) (132), targeted sequencing (135), and chromosomal genomic array testing (CGAT) (129). Sequencing of relapsed blasts may also identify new mutations, revealing potential targets for salvage therapy. With bioinformatic tools such as sciClone and fishplot (132, 135), serial samples collected before and after CAR-T cell therapy can be used to reconstruct clonal evolution, mapping the emergence of subclones and newly acquired mutations (132, 135).

Where feasible, single-cell technologies provide even finer resolution. Single-cell DNA sequencing (scDNA-seq) and scRNA-seq can outline clonal trajectories and help pinpoint founder clones for targeted intervention. For example, Tapestri Insights with scDNA-seq enables inference of clonal architecture and tracking of mutation dynamics during treatment (135). Similarly, scRNA-seq-based trajectory analysis can define developmental stages and cell clusters involved in lineage-switch reprogramming (135) or leukemia initiation (137).

Sequencing of B-cell receptor (BCR), immunoglobulin heavy chain (IGH) or V(D)J regions is also widely used for clonal analysis (129, 135, 136). Here, differences between primary and relapse samples are often more informative than shared sequences, as they can clarify disease evolution and the likely cellular origin of lineage-switch relapse. Lack of shared BCR/IGH/V(D)J rearrangements between lineage-switch relapse and the preceding ALL—or complete absence of such rearrangements in the relapse—suggests origin at an early developmental stage (145), such as an immature stem cell clone, as reported by Gardner et al. (129). If the lineage-switch relapse carries unrelated rearrangements, this suggests that the relapsed myeloid blasts either harbor aberrant rearrangements, arise from B-lymphoid cells already committed to rearrangement, or are reprogrammed from a minor pre-existing ALL clone (145). Conversely, when lineage-switch relapse and the original ALL share rearrangements, the myeloid cells are likely derived through direct transdifferentiation or reprogramming from the major ALL clone (145).

#### 3.1.5 Therapeutic strategies for different types of relapses

Relapse phenotypes can be defined using the assessments described above and treated accordingly. Overall, outcomes after relapse from CD19 CAR-T therapy are poor, with a median survival of 7.5 months (154). Nevertheless, several therapeutic options are available. Notably, high-intensity chemotherapy and HSCT remain broadly applicable and will not be repeatedly detailed, unless specified otherwise. In addition, HSCT may be considered after any salvage regimen, although prognosis remains unfavorable (154).

For CD19^+^ relapse with a persistent B-cell phenotype, CD19 CAR-T retreatment is a reasonable option, which typically requires a high proportion of CD19^+^ blasts (usually ≥90%) (4, 9, 10, 12, 13). However, responses vary by cases. In ZUMA-5, all 13 patients with relapsed lymphoma responded (7 complete remission/CR, 6 partial remission/PR) (14), whereas only 40% of patients with relapsed B-ALL responded (all CR) with a second infusion in study conducted by Wudhikarn et al. (154). Shah et al. (18) observed no CRs in patients with relapsed B-ALL after retreatment in their cohort. Gardner et al. (21, 22) also reported frequent CAR-T engraftment failure upon retreatment in relapsed B-ALL. Comprehensive reassessment of relapsed blasts (including lineage bias, CD19 expression level, and new mutations) is therefore crucial before considering another CAR-T infusion.

Additionally, blinatumomab is an effective salvage therapy for CD19^+^ relapse (1, 154, 158), including cases with low CD19 expression (158). Qi et al. (158) reported that 4 of 5 relapsed B-ALL patients responded and were alive at the cutoff date (3 in CR/CRi); the single death was due to post-HSCT infection. Wudhikarn et al. (154) also reported encouraging responses, though in a small cohort.

For CD19^-^ relapse with preserved B-cell phenotype, Inotuzumab ozogamicin, a CD22-directed antibody-drug conjugate, is commonly used and also applicable to CD19^+^ relapse, as demonstrated in the ELIANA trial (1). CD22 expression, a key parameter in relapse blast assessment (section 3.1.3), guides its use. Inotuzumab ozogamicin is active in B-ALL relapsing after CAR-T therapy, but durability is limited. Li et al. (159) reported CR in all three heavily treated r/r B-ALL patients relapsing after CD19 CAR-T and consolidative HSCT. Although all three patients survived after one year of treatment, 2 of them relapsed. Similarly, Wudhikarn et al. (154) observed a CR rate >50%, but with only one long-term survivor.

Lineage switch relapse is particularly challenging, with high heterogeneity and poor outcomes. In Project EVOLVE (lineage switch after CAR-T or other immunotherapies) (127), the most common strategies were intensive AML-induction-type chemotherapy (41.5%) and CD33-targeted gemtuzumab ozogamicin (21.5%), highlighting the value of detailed immunophenotyping. These two strategies are most effective as well: either high-dose chemotherapy alone or combined with gemtuzumab achieved a 30.4% CR rate (127). Given the AML-like biology, venetoclax is also appropriate for lineage switch relapse. Although sparsely used with limited overall efficacy in Project EVOLVE (127), several post-CAR-T lineage switch relapse cases achieved, and in some instances maintained, remission with venetoclax-based regimens (133, 135, 136). In addition, consolidative HSCT after first-line therapy is recommended and was used in 21.5% of patients in Project EVOLVE cohort (127). However, despite the acceptable CR rate of the first line therapy and the subsequent consolidative HSCT, the long-term survival of lineage switch still needs improving: only 8 of 69 patients were alive at the cutoff (127).

In addition to chemotherapy, HSCT and general therapies, sequencing approaches and assessments can reveal additional, patient-specific targets. Liu et al. (136) identified a persistent BCR::ABL1 fusion in AML relapse arising from Ph^+^ B-ALL after CAR-T; treatment with dasatinib plus venetoclax followed by dasatinib maintenance led to ongoing CR (136). Fusion status, however, may change, evidenced by another post-CAR-T AML relapse from Ph^+^ B-ALL who lost BCR::ABL1 at relapse (134). FLT3::ITD mutations, either persistent (135) or newly acquired (134), have also been reported in lineage switch relapses after CAR-T therapy, supporting early use of FLT3 inhibitors such as gilteritinib. (In these two cases, FLT3 inhibitors were not used; both patients achieved CR with chemotherapy alone.) In addition, agents targeting KMT2A rearrangements are in development (160, 161). Although single-agent efficacy in lineage switch remains uncertain (127), they are promising given the high frequency of KMT2A rearrangements in this setting.

Serial sequencing can also identify key survival pathways in relapse blasts, providing potential candidates for therapeutic targeting. Cui et al. (137) reported marked activation of the Annexin A1 (ANXA1)-FPR1/2 pathway after lineage switch following CAR-T in a patient who died from relapse despite chemotherapy. MDX-124, an anti-ANXA1 monoclonal antibody now in clinical trials (162), may represent a future option for lineage switch relapses driven by ANXA1-FPR1/2 signaling.

#### 3.1.6 Potential lineage reversion back to B-ALL and the role of epigenetic regimens

A case report (163) and Project EVOLVE (127) have described rare reversions of myeloid relapse back to the original B-ALL phenotype after myeloid-directed therapy. Such reversion was more frequent in partial lineage switch (partial loss of B-cell markers or gain of myeloid markers), with 5 of 10 relapses reverting to B-ALL, compared with a 10.8% reversion rate in complete lineage switch (127). These findings highlight the need to monitor blast phenotype at relapse and to adjust treatment promptly once lineage reversion occurs.

Although based on limited numbers, lineage reversion to B-ALL appears to be associated with better outcomes. In Project EVOLVE, 3 of 7 patients with lineage reversion were alive (2 in CR), whereas only 5 of 62 patients with lineage switch without reversion survived (127). Unlike other immunotherapies in Project EVOLVE, which have short-lived antitumor activity, CAR-T cells can persist for prolonged periods. Post-CAR-T CD19^-^ relapse often arises while CD19-CAR-T cells remain functional, so restoring CD19 expression can re-sensitize blasts to CAR-T-mediated killing. Thus, despite the need for more data, forced CD19 re-expression or induced lineage reversion is an attractive strategy for lineage switch relapse after CAR-T therapy.

Because clinical experience with lineage reversion after post-CAR-T lineage switch is limited, the rationale for CD19 re-expression/lineage reversion therapy relies largely on mechanistic insights. As discussed in Section 2.3.4, lineage switch after CAR-T therapy may result from mutations in key regulators (e.g., PAX5, EBF1), persistence of bipotent progenitors, or epigenetic dysregulation. When pathogenic mutations in master regulators are present, lineage reversion is unlikely, as restoring normal gene function is currently infeasible. In leukemias driven by bipotent progenitors, myeloid-directed therapy may exert selection pressure that favors a shift back to the lymphoid lineage, allowing elimination by CAR-T cells or other B-ALL-directed regimens, although long-term remission still requires eradication of these progenitors. Epigenetically, lineage switch relapse is linked to aberrant H3K27 acetylation (149), chromatin remodeling (145) and potential PAX5 hypermethylation, suggesting that the myeloid phenotype or its survival may depend on specific, targetable epigenetic states. If no obvious key gene mutations or bipotent progenitors are identified, epigenetic profiling (e.g., DNA methylation analysis) of relapse samples is advisable. Epigenetic-modifying agents may then help eradicate relapsed blasts or drive lineage-switched cells back to their original B-ALL phenotype, restoring sensitivity to CAR-T cells. As summarized in **Table 2**, the DNA methyltransferase inhibitors decitabine and azacitidine have induced CR and long-term survival in several reported cases of lineage switch relapse (134, 135).

Besides lineage switch relapse, the concept of forced CD19 re-expression may also apply to other forms of CD19^-^ relapse. It is not appropriate for relapses arising from pre-existing CD19^-^ clones or irreversible CD19 lesions, but CD19 expression is at least partly plastic, and its loss can be reversible, as shown in murine B-lymphoma models (164). As described in Section 2.2.2.1, epigenetic mechanisms contribute to CD19 downregulation in CD19^-^ relapse, and the demethylating agent 5-aza-2’-deoxycytidine can restore CD19 expression in a murine CD19^-^ relapse model (109). Guided by epigenetic profiling, forced CD19 re-expression therefore represents a theoretically promising approach for treating CD19^-^ relapse after CAR-T cell therapy.

### 3.2 Solution and future perspectives

Current strategies for managing CD19^-^ relapse remain inadequate, and substantial work is needed to translate preclinical advances into effective clinical options. Still, several emerging approaches offer promise. A direct approach after CD19 CAR-T failure is to target alternative antigens. CAR-T cells recognizing CD22 or CD20, alone or in combination with CD19, can partially overcome escape due to CD19 loss (165–170). However, CD22 is co-downregulated with CD19 (110) (although contradictory result exists (171)), and CD20 is frequently expressed at low levels (172). Although CD22^−/low^ relapse is less common (170), CD22 CAR-T cell efficacy depends on CD22 density, with CD22^−^ relapses still occurring (168). Even tandem CD22/CD19 CAR-T cells do not fully clear relapsed CD19^−^ cells after CD19 CAR-T treatment (96). These limitations motivate the exploration of new targets. Leukocyte-immunoglobulin-like-receptor-B1 (LILRB1) is highly expressed in B-ALL, B-cell lymphoma and AML, even after CD20/CD19-directed immunotherapies, making LILRB1 CAR-T cells a potential salvage option for CD19^−^ relapse and lineage switch (173). CD123, which is enriched on leukemia progenitors and CD19^−^ relapse, is another attractive target and may help prevent CD19^−^ relapse after CD19-directed therapy (174). Such ability of targeting progenitors is particularly important for CD19^−^ lineage switch relapse caused by bipotent progenitors. B-cell activating factor receptor (BAFF-R), with similar expression patterns, represents an additional candidate CAR target (175).

Additionally, our proposed comprehensive assessment workflow (Section 3.1.3) enables identification of pre-existing CD19^−^ subclones and those with myeloid potential. This information can guide personalized therapies targeting antigens on these subclones, aiming to eradicate the progenitor/initiating pool of CD19^−^ relapse. Bispecific CAR-T cells co-targeting these subclones and CD19 could further reduce the risk of subsequent CD19^−^ relapse.

Besides exploring novel antigens, several strategies also focus on the relapsed CD19^−^ blasts themselves. CD19^low^ resistant cells after CAR-T cell therapy are highly sensitive to Bruton’s tyrosine kinase (BTK) and MEK inhibition, a finding supported by Im et al. (116), suggesting a pharmacologic salvage strategy. Bortezomib and mitoxantrone also show *in vitro* efficacy against CD19^−^ B-ALL cell lines through AKT and mTOR inhibition (176). Restoring CD19 expression is another salvage option (Section 3.1.6). Notably, upon a change of therapy and relief of selective pressure, relapsed CD19^−^ blasts in most patients restore CD19 expression (85), although not all studies confirm this (177). Because mechanisms of CD19 downregulation are heterogeneous, decisions about forced CD19 re-expression should be guided by comprehensive profiling, including epigenetic assessment.

CD19^−^ relapse after CD19 CAR-T therapy is biologically complex and highly heterogeneous. Different CAR designs impose distinct selective pressures, driving diverse CD19 alterations, while host genetics further shape tumor evolution. Advances in cutting-edge technologies now enable more precise characterization of CD19 modulation under immune pressure. Sequencing tools discussed in Section 3.1 can be applied to both pre- and post-CAR-T blasts for lineage and clonal analysis. Single-cell methods can reveal pre-existing CD19^−^ subclones and progenitor-like populations. However, the limited sequencing depth of single-cell technologies may compromise their ability to detect CD19 isoforms and other genomic alterations. In lineage-switch relapse, single-cell analysis can define unique markers suitable for targeted therapy. Deep genomic sequencing can uncover genomic vulnerability such as PAX5 SNPs or pre-existing CD19 mRNA variants that may predict CD19^−^ relapse risk. Long-read sequencing can resolve CD19 isoform structures, informing CAR designs that target critical domains. Surface proteomics can systematically identify additional surface antigens for CAR-T targeting.

Beyond conventional T cells, other CAR-engineered immune cells maintain intrinsic effector functions and may kill CD19^-^ blasts regardless of the specific mechanism of CD19 loss (178). Armored CAR-T cells are another promising avenue. Fourth-generation CARs, or T cell redirected for universal cytokine-mediated killing (TRUCKs), secrete proinflammatory cytokines, either constitutively or upon activation, to boost endogenous immunity and remodel the tumor microenvironment (179). In murine lymphoma models, IL-12-expressing CD19 CAR-T cells elicit strong host immune responses and durable survival without preconditioning (180, 181). CAR-T cells engineered to express IL-18 similarly enhance anticancer immunity (182, 183). Clinically, CAR-T cells secreting IL-18 (huCART19-IL18) has produced high response rates in patients with r/r lymphoma after failure of prior anti-CD19 CAR-T therapy (81% overall response; 52% CR) (184).

Ultimately, persistent selective pressure will continue to drive immune escape. This arms race between cancer and the immune system underscores the need for sustained, mechanistically informed innovation to achieve more durable control of CD19^−^ relapse.
